# Prevention of Vascular Calcification by Magnesium and Selected Polyphenols

**DOI:** 10.1155/2021/6686597

**Published:** 2021-04-09

**Authors:** Haile Mehansho, Satya Majeti, Gabe Tzeghai

**Affiliations:** Summit Innovation Labs, 1776 Mentor Avenue, Cincinnati, OH 45212, USA

## Abstract

Arterial vascular calcification (VC) represents formation of calcium phosphate deposits on the interior of arteries, which could restrict blood flow leading to heart health problems, including morbidity and mortality. VC is a complex and tightly regulated process that involves transformation of vascular smooth muscle cells (VSMCs) to bone-like cells and subsequent deposition of calcium as hydroxyapatite. Natural bioactives, including quercetin (Q), curcumin (C), resveratrol (R), and magnesium (Mg), have been reported to inhibit VC. Thus, we conducted an in vitro study using rat vascular smooth muscle cells (rVSMCs) to evaluate the protective effect of natural bioactives found in OptiCel, that is, Mg combined with polyphenols (PPs), Q, C, and R. Calcification was induced by culturing rVSMCs in a high phosphate (HP) medium. The addition of Mg and Q + C + R separately decreased the HP-induced calcium deposition by 37.55% and 42.78%, respectively. In contrast, when Mg was combined with Q, C, and R, the inhibition of calcium deposition was decreased by 92.88%, which is greater than their calculated additive inhibition (80.33%). These results demonstrate that the combination of Mg with selected PPs (Q, C, and R) is more effective than when used separately. The findings also suggest the combination has a synergistic effect in inhibiting VC, which is a risk factor for cardiovascular disease. Thus, regular consumption of these natural bioactives could have a beneficial effect in reducing the development of heart diseases.

## 1. Introduction

Despite numerous medications and intervention procedures, cardiovascular disease (CVD) continues to be a major cause of morbidity and mortality. In fact, CVD is the most common cause of death in the world and accounts for approximately one-third of all deaths [1]. In 2017, there were nearly 650,000 deaths in the U.S, due to CVD, which accounted for 23% of all deaths [2].

The primary cause of heart disease is the hardening of the artery, which is known as atherosclerosis [3, 4]. Under this condition, fat and calcium build up in the wall of the arteries and form a hard structure called plaque [3]. The resulting calcified plaque causes the arteries to become narrower and stiff and reduces and/or blocks blood flow to the various organs, which subsequently leads to heart attack and stroke. Vascular calcification (VC) in the vascular smooth muscle cells (VSMCs), which is characterized by the deposition of calcium phosphate in the form of hydroxyapatite, is recognized as one of the major risk factors for the development and progression of heart diseases [3–6].

The development of VC has been shown to be a tightly regulated metabolic process [5, 6]. It involves the differentiation of normal contractile phenotype VSMCs to osteogenic/bone-like cells and deposition of calcium as hydroxyapatite [5, 6]. Multiple triggers are involved in the development of VC. This includes oxidative stress, inflammation, hypertension, diabetes, hyperphosphatemia, kidney disease, dyslipidemia, high calcium, and high vitamin D [5, 6]. These triggers induce VC by upregulating the expression of various transcription factors, signaling pathways, and growth factors, which promote differentiation of VSMCs to bone-like cells and subsequent deposition of calcification as hydroxyapatite [5–9].

Currently, there is no treatment available for VC even though it is one of the major risk factors for the development of heart disease. Natural compounds, commonly found in fruits, vegetables, spices, and herbs, have been shown to inhibit VC development by suppressing differentiation of VSMCs to osteogenic/bone-like cells and formation of calcium as hydroxyapatite [10–14]. Magnesium (Mg) and quercetin (Q) commonly found in fruits and vegetables [15, 16], resveratrol (R) contained in grapes and berries [17], and curcumin (C) obtained from the spice turmeric [18] have been shown to inhibit VC in a number of studies [10–14].

To date, there are no reported studies on the effect of Mg combined with selected PPs (Q, C, and R) on VC. Thus, the objective of our study is to evaluate the effect of Mg and in combination with C, Q, and R on VC in rVSMCs, that is, bioactives currently used in OptiCel Heart Health dietary supplement. Specifically, the aim of this research is to assess additive and potentially synergistic benefits from the bioactives in preventing vascular calcification.

## 2. Materials and Methods

### 2.1. Cell Culture

rVSMC primary cells (Lonza R-ASM-580; 3001900115) were cultured following the procedures described by others with slight modifications [12, 14]. Cells were cultured in a humidified atmosphere at 37°C and 5% CO_2_. Standard culture medium consisted of DMEM (Gibco 1 1966- 500), streptomycin/penicillin (Gibco 1 5630-080), and 0.3% DSMO (Sigma-Aldrich D2650), supplemented with 20% fetal bovine serum (FBS, SERADIGM 1 500-500). Cell layers were detached with 0.05% trypsin/0.02% EDTA solution (GIBCO 25200). Cells were maintained at 70–80% confluence by passing as needed. A portion of the rVSMCs was stored for later use under frozen conditions using 90% FBS (Seradigm 1 500-500) and 10% DMSO (Sigma D2650).

### 2.2. Experimental Design

In the control group (CL), rVSMCs were cultured in a standard culture medium, which contains physiological magnesium concentration (0.8 mM) and phosphate concentration (0.9 mM). Calcification was induced by incubating rVSMCs in the high phosphate (HP) medium (5.0 mM). To increase the phosphate concentration, a combination of disodium hydrogen phosphate (Na_2_HPO_4_ EMD SX0720-1) and sodium dihydrogen phosphate (NaH_2_PO_4_ EMD SX0710-1) at 1 : 2 ratio was added. To evaluate the inhibition of VC by the bioactives, Q (Tocris 1125) at 50 *µ*M, R (Tocris 1418/100)) at 100 *µ*M, and C (Sigma-Aldrich, C7727) at 5 *µ*M were added to the HP medium. Mg was added as magnesium chloride (Sigma R0971) to increase the final magnesium concentration to 1.4 mM (Mg). The treatment groups in the study included the following: (a) CL, (b) HP, (c) Mg, (d) Q + C + R, and (e) Mg + Q + C + R. All bioactives were dissolved in 0.3% DMSO media. The concentrations for each bioactive were chosen based on previous in vitro studies with slight modifications [10–13].

### 2.3. Induction of Calcification and Treatment with Mg, Q + C + R, and Combination of Mg + Q + C + R in rVSMCs

Cells were thawed and allowed to grow in a growth medium for 9 days in a 37°C, 5% CO_2_ incubator. Then, they were dispensed into a 96-well plate at 20,000 to 60,000 cells per well. After the cells were adhered overnight, the medium was replaced with growth medium alone for the CL group or calcification medium, 5 mM phosphate with and without the addition of the bioactives. The rVSMCs in the different treatments were cultured for 9 days with fresh media and exchanged every three to four days. Each treatment was tested in four replicates.

### 2.4. Analysis of VSMC Calcification

After 9 days of incubation, the medium was removed, and the monolayer cells were washed twice with phosphate-buffered saline (PBS). Then, the cells were decalcified overnight with 0.6 N HCl at 2–8°C, and the supernatant was analyzed for calcium content by using the QuantiChrom™ Calcium Assay Kit (BioAssay Systems, CA, USA) following the instructions provided by the manufacturer.

### 2.5. Statistical Analyses

Data are expressed as mean ± SD. The *T*-test was used to determine the difference between treatment means by using Microsoft Excel. A *P* value <0.5 was considered statistically significant.

## 3. Results

### 3.1. Induction of Calcification in rVSMCs by HP

To induce calcification, the phosphate concentration in the HP group was increased from 0.9 mM to 5 mM. At the end of 9 days of incubation period, the calcium content of the HP group was 8.49 ± 2.57 *µ*g/100 *µ*l and 0.29 ± 0.16 *µ*g/100 *µ*l for the CL group (*p* < 0.001) (Table 1).

### 3.2. Inhibition of rVSMC Calcification by Mg and Q + C + R

**Figure 1:**
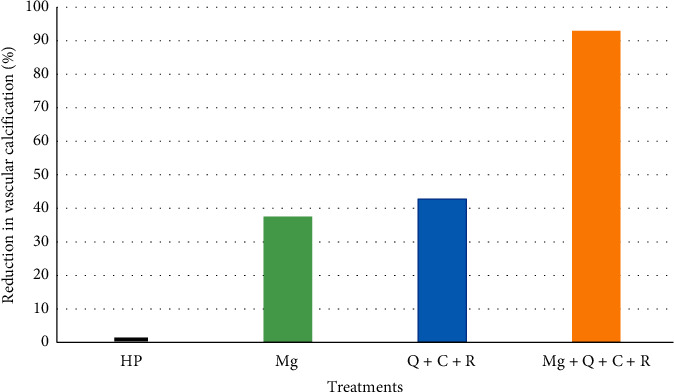
Percent reduction of vascular calcification in rVSMCs incubated in the HP medium by Mg, Q + C + R, and Mg + Q + C + R.

After 9 days of incubation, increasing the concentration of Mg to 1.4 mM decreased calcification from 8.49 ± 2.57 *µ*g/100 *µ*l in the HP group to 5.30 ± 0.78 *µ*g/100 *µ*l (*p* < 0.05) (Table 1). Similarly, addition of polyphenols combination, Q + C + R, reduced HP-induced calcification from 8.49 ± 2.57 *µ*g/100 *µ*l to 4.86 ± 0.28 *µ*g/100 *µ*l (*p* < 0.01) (Table 1). Addition of Mg and Q + C + R decreased the high phosphate-induced calcium deposition by 37.55% and 42.78%, respectively (Figure 1).

### 3.3. Effect of Mg and Q + C + R Combination in Inhibiting Calcification in rVSMCs

Addition of the Mg and selected polyphenols (Q + C + R) combination decreased calcification from 8.49 ± 2.57 *µ*g/100 *µ*l in the HP group to 0.60 + 0.21 *µ*g/100 *µ*l (*p* < 0.001) (Table 1). The combination of Mg and Q + C + R reduced calcification by 92.88% (Figure 1). This reduction in calcification by the combination of Mg + Q + C + R was greater than the calculated additive effect of Mg alone and Q + C + R alone (80.33%).

## 4. Discussion

Diets, particularly those rich in fruits, vegetables, and spices, have been shown to have strong association with the reduction in VC [18]. The health benefits of these foods are attributed to the various bioactives, including minerals, vitamins, and phytonutrients [15–17, 19].

In this study, we have evaluated the effect of Mg with and without the polyphenols (Q + C + R), which are naturally found in fruits, vegetables, and spices, in an in vitro study using rVSMCs. Mg alone reduced the increased phosphate-induced calcification by 37.55%. This value is in agreement with the results previously reported by others [12, 20, 21]. Also, addition of Q, C, and R in combination with the high phosphate medium decreased VC by 42.78%. However, the treatment with Mg and Q + C + R combination inhibited the calcification of rVSMCs by 92.88%. This combined effect of Mg and the polyphenols is greater than the calculated additive effect of Mg and Q + C + R (80.33%). The results suggest the combination of Mg and the polyphenols, Q, C, and R, has a synergistic effect in preventing VC. To the best of our knowledge, this is the first study that demonstrated almost complete inhibition of VC, when Mg and polyphenols, Q, C, and R, are used in combination.

Multiple studies have shown Mg, Q, C, and R are effective in inhibiting calcification in VSMCs, when tested individually [10–13]. The results from an in vitro study showed quercetin is effective in inhibiting a warfarin-induced VC by inhibiting the differentiation of VSMCs to osteoblastic-like cells by suppressing the expression of *β*-catenin signaling pathway [11, 22]. Using similar in vitro studies, Mg has been shown to inhibit phosphate-induced VC by suppressing the differentiation of VSMCs to bone-like cells and formation of hydroxyapatite [12, 20, 21]. The inhibition of VC by Mg was shown to be mediated through suppressing the expression of growth factors, bone morphogenic proteins, osteogenic transcription factors (runt-related transcription factor-2, and osterix [12, 20, 21]. Resveratrol has been shown to inhibit calcification of VSMCs by activating sirtuin 1 (SIRT1), an inhibitor of both Runx2 and *β*-catenin signaling [13, 22, 23]. Roman-Garcia et al. [10] also demonstrated an addition of curcumin reduces a calcium plus phosphate-induced VC by inhibiting the differentiation of VSMCs to bone-like cells by suppressing the expression of Runx2 [10].

The development of VC is a complex metabolic process. The two major and critical steps involved in the development of VC include the transformation of normal VSMCs to osteogenic-like cells and calcium deposition in the form of hydroxyapatite crystals [2, 3]. These metabolic processes are regulated by multiple inducers, which include transcription factors, growth factors, and signaling pathways [5–9], and inhibitor proteins such as osteopontin, osteoprotegerin, and matrix Gla protein [2, 3]. Although further study is needed, the data clearly indicate that the combination of Mg + C + Q + R provides at least an additional benefit. These data also suggest that the combination is likely providing synergistic benefits by modulating either the same inducers and inhibitors or perhaps additional others that are involved in the development of VC [10–13, 20–23].

## 5. Conclusion

The results from our study demonstrate the addition of magnesium and Q, R, and C combination almost completely inhibited the development of phosphate-induced vascular calcification in rVSMCs. It is likely that the combination of Mg and selected polyphenols is having a synergistic effect in inhibiting VC by downregulating the multiple triggers, pathways, and mechanisms involved in the development of VC. Even though the study's finding needs to be corroborated with clinical data, it does suggest consumption of magnesium, quercetin, curcumin, and resveratrol together could more effectively contribute to the prevention and treatment of heart disease by inhibiting the development and progression of vascular calcification.

## Figures and Tables

**Table 1:** Effect of Mg, Q + C + R, and Mg + Q + C + R in rVSMCs cultured in the HP medium.

Treatments	Average Ca content (*µ*g/100 *µ*l ± SD)
CL	0.29 ± 0.16
HP	8.49 ± 2.57^a^^*∗∗∗*^
Mg	5.30 ± 0.78^b^^*∗*^
Q + C + R	4.86 ± 0.28^b^^*∗∗*^
Mg + Q + C + R	0.60 ± 0.21^b^^*∗∗∗*^

In the control group (CL), VSMCs were cultured in a medium containing 0.9 mM phosphate and 0.8 mM magnesium. In the treatment groups, high phosphate (HP) concentration was increased from 0.9 mM to 5 mM (HP). In the Mg group, the Mg level was increased from 0.8 mM to 1.4 mM. The polyphenol concentrations used were 50 *μ*M for Q, 5 *μ*M for C, and 100 *μ*M for R. The results are presented as mean ± SD of four replicates. ^a^*p* values as compared with CL, ^b^*p* values as compared with HP, and ^c^*p* values as compared with Mg. ^*∗*^*p* value < 0.05; ^*∗∗*^*p* value < 0.01; ^*∗∗∗*^*p* value < 0.001.

## Data Availability

Key data are included in the article. Data on a similar study and outcome are available from Dr. Gabe Tzeghai.
